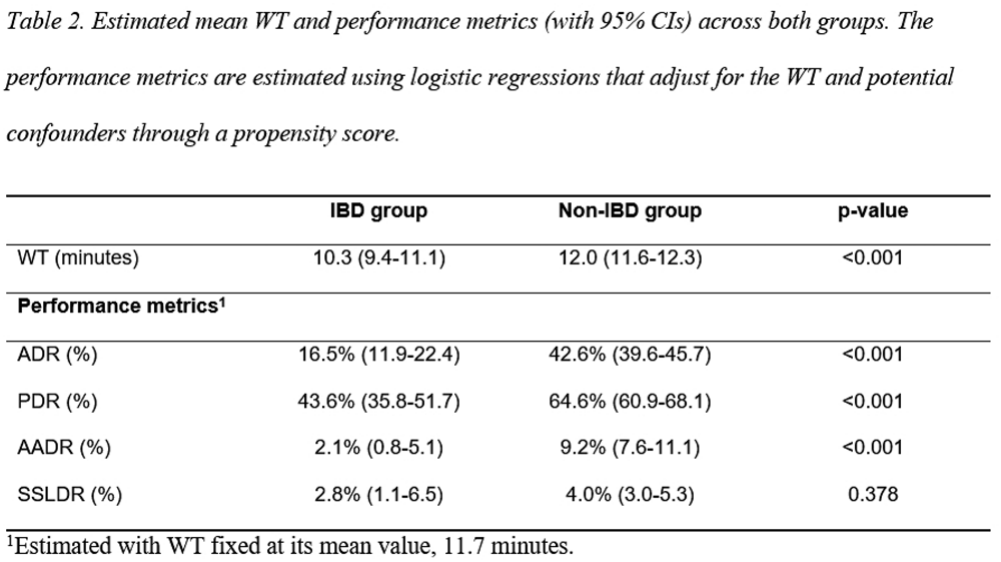# Poster Session I - A51 COGNITIVE LOAD AND ADENOMA DETECTION IN IBD SURVEILLANCE COLONOSCOPY: A PROSPECTIVE STUDY

**DOI:** 10.1093/jcag/gwaf042.051

**Published:** 2026-02-13

**Authors:** C G Ingabire, R Battat, D K Rex, D C Daoud, E Bernard, K Orlicka, R Leduc, L D’aoust, V Michal, M Oleksiw, R Djinbachian, E deslandres, M Bouin, J Liu, Chen Kiow, P Benoit, S Bouchard, D Von Renteln

**Affiliations:** Centre de Recherche du Centre Hospitalier de l’Universite de Montreal, Montreal, QC, Canada; Centre Hospitalier de l’Universite de Montreal, Montreal, QC, Canada; Indiana University School of Medicine, Indianapolis, IN; Centre Hospitalier de l’Universite de Montreal, Montreal, QC, Canada; Centre Hospitalier de l’Universite de Montreal, Montreal, QC, Canada; Centre Hospitalier de l’Universite de Montreal, Montreal, QC, Canada; Centre Hospitalier de l’Universite de Montreal, Montreal, QC, Canada; Centre Hospitalier de l’Universite de Montreal, Montreal, QC, Canada; Centre de Recherche du Centre Hospitalier de l’Universite de Montreal, Montreal, QC, Canada; Centre de Recherche du Centre Hospitalier de l’Universite de Montreal, Montreal, QC, Canada; Centre Hospitalier de l’Universite de Montreal, Montreal, QC, Canada; Centre Hospitalier de l’Universite de Montreal, Montreal, QC, Canada; Centre Hospitalier de l’Universite de Montreal, Montreal, QC, Canada; Centre Hospitalier de l’Universite de Montreal, Montreal, QC, Canada; Centre Hospitalier de l’Universite de Montreal, Montreal, QC, Canada; Centre Hospitalier de l’Universite de Montreal, Montreal, QC, Canada; Centre Hospitalier de l’Universite de Montreal, Montreal, QC, Canada

## Abstract

**Background:**

Colonoscopy is central to colorectal cancer (CRC) prevention, with its quality assessed by adenoma detection rate (ADR) and process measures like withdrawal time (WT). In inflammatory bowel disease (IBD) surveillance, the procedural and cognitive workload is higher, and IBD-specific tasks consume WT minutes. Yet, current guidelines provide no IBD-specific WT or ADR benchmark, and average-risk thresholds are routinely extrapolated to this cohort who faces twice the risk of CRC. Given the close link between WT and ADR, and consistent reports of lower IBD ADRs, concerns arise that standard WT targets are insufficient, and screening colonoscopy quality may be suboptimal in IBD patients.

**Aims:**

Our primary aim was to evaluate colonoscopy quality by comparing ADR in screening-age IBD patients to non-IBD patient under a standardized WT. It was hypothesized that for equal WTs, the ADR in non-IBD cases would be higher than the one in IBD cases, and that longer WT would be necessary for colonoscopies performed on IBD patients to reach similar ADR levels as in non-IBD colonoscopies

**Methods:**

We conducted a prospective observational study, using a video library of endoscopic procedures from our tertiary care center. All consecutive patients ≥45 years who underwent complete colonoscopy (2023-2025) were included. The primary outcome compared ADR between IBD and non-IBD groups, with adjustment for WT. Secondary outcomes assessed mean WT, polyp detection rate (PDR), advanced adenoma detection rate (AADR), and sessile serrated lesion detection rate (SSLDR) across both groups.

**Results:**

We included 1507 colonoscopies (241 IBD, 1266 non-IBD). When WT was held constant at 11.7 minutes, the IBD group’s ADR (16.5%) was significantly lower than non-IBD (42.6%). ADR increased considerably faster with WT in non-IBD patients (16.8% vs 4.73% per minute). Achieving a 26% ADR required in 6.9 minutes of WT for non-IBD cases compared to 24.2 minutes for IBD. Furthermore, IBD colonoscopies had shorter mean WT (10.3 min vs 12.0 min).

**Conclusions:**

ADR was considerably lower in IBD compared to non-IBD patients and increased far more slowly with additional WT in IBD. This suggest that a significant portion of procedural time and cognitive focus is devoted to IBD-specific tasks, thereby limiting mucosal observation for CRC precursors. Together, this shows that the current 8-minute WT target is insufficient for IBD surveillance and that average-risk thresholds are poorly suited to this high-risk cohort. We therefore recommend establishing IBD-specific quality indicators, including a tailored WT benchmark, to optimize CRC prevention strategies in this vulnerable population.

**Funding Agencies:**

None